# Cytotoxicity and Immunomodulatory Effects of Cannabidiol on Canine PBMCs: A Study in LPS-Stimulated and Epileptic Dogs

**DOI:** 10.3390/ani14243683

**Published:** 2024-12-20

**Authors:** Phannicha Kosukwatthana, Oumaporn Rungsuriyawiboon, Jatuporn Rattanasrisomporn, Kanogwan Kimram, Natthasit Tansakul

**Affiliations:** 1 Graduate Program in Animal Health and Biomedical Sciences, Faculty of Veterinary Medicine, Kasetsart University, Bangkok 10900, Thailand; phannicha.ko@ku.th; 2 Department of Veterinary Technology, Faculty of Veterinary Technology, Kasetsart University, Bangkok 10900, Thailand; cvtopr@ku.ac.th; 3 Department of Companion Animal Clinical Sciences, Faculty of Veterinary Medicine, Kasetsart University, Bangkok 10900, Thailand; fvetjpn@ku.ac.th; 4 Kasetsart University Veterinary Teaching Hospital, Faculty of Veterinary Medicine, Kasetsart University, Bangkok 10900, Thailand; drkimgolf@gmail.com; 5 Department of Pharmacology, Faculty of Veterinary Medicine, Kasetsart University, Bangkok 10900, Thailand

**Keywords:** cannabidiol, CBD, epilepsy, cytokines, canine

## Abstract

Cannabidiol, the primary non-psychoactive phytocannabinoid found in cannabis, has generated significant research interest due to its potential for biological effects, such as anti-inflammatory, analgesic, immunomodulatory, and anticonvulsant properties. Several studies have demonstrated the potential of CBD to alter inflammatory cytokines; however, data on CBD’s effects on cell viability and pro-inflammatory cytokines in target animals, such as dogs, are limited. Therefore, in this study, we investigated the effects of CBD on the cell viability and modulation of pro-inflammatory cytokines, such as interleukin (IL)-1β, IL-6, and tumor necrosis factor-α (TNF-α), in canine PBMCs stimulated with LPS. To evaluate the effect of CBD on neuroinflammation in epilepsy pathology, an independent study of five refractory epileptic dogs co-treated with CBD for 30 days was conducted. The current findings revealed that CBD concentrations of 16 µg/mL had a statistically significant effect on the viability of canine PBMCs with a calculated IC_50_ of 15.54 µg/mL. The effect of CBD on inflammatory cytokines in LPS-stimulated PBMCs tended to be dose-dependent, with CBD concentrations of 5–30 μg/mL resulting in decreased production of the tested pro-inflammatory cytokines. Considering the effect of CBD on cytokine production by PBMCs from epileptic dogs, CBD has the potential to modulate immune responses and provide benefits when used in combination with antiepileptic drugs. The findings provided evidence of CBD cytotoxicity and its effect on the alteration of pro-inflammatory cytokines in canine PBMCs.

## Simple Summary

Cannabidiol has gained attention owing to its potential anti-inflammatory and neuroprotective properties. This research examined cannabidiol’s cytotoxicity and its effects on cytokines in lipopolysaccharide-activated peripheral blood mononuclear cells from both healthy canines and those with idiopathic epilepsy. The study’s findings indicate that cannabidiol may have a role in modulating pro-inflammatory cytokine levels, suggesting potential therapeutic applications in various medical conditions.

## 1. Introduction

Cannabidiol, a non-psychotropic component of cannabis, is of increasing interest in various research domains due to its potential to treat diseases or serve as a supplement for both human and animal health [1]. CBD has been found to interact with the endocannabinoid system and exert biological effects in several species [2]. Notably, the endocannabinoid system may be a component of therapeutic strategies for inflammatory and neuropathic pain conditions [3]. Although the mechanism of action of CBD is not yet clearly elucidated, there exists substantial anecdotal evidence and clinical efficacy of CBD utilization [4,5]. In veterinary medicine, several reports have demonstrated the pharmacological effects of CBD, including anti-inflammatory, analgesic, dermatological, and immunomodulatory properties [6,7,8,9].

Given the extensive range of CBD products utilized in human and animal populations, there is increasing interest in its potential to mitigate inflammation, pain, and neurological disorders. Animals with inflammatory and pain conditions, such as osteoarthritis, have demonstrated improvements in pain management and mobility when treated with CBD, potentially due to its anti-inflammatory or nociceptive properties [7,10]. Several studies have indicated that CBD may contribute to the reduction of neuroinflammation and provide neuroprotective effects [11,12]. Animal studies and preliminary human trials have yielded promising results in conditions such as multiple sclerosis and traumatic brain injury [13,14].

The inflammatory response is a complex biological response of the immune system to harmful pathological conditions. This response process is primarily driven by hierarchical cascade interactions among the inflammatory mediator network. Pro-inflammatory and anti-inflammatory cytokines are essential mediators in regulating the growth and communication between other cells in the immune system and are predominantly produced by T cells and macrophages [15,16]. Peripheral blood mononuclear cells (PBMCs), which include lymphocytes, macrophages, and dendritic cells, are involved in immune-related functions. The pro-inflammatory cytokines, including interleukin-1 beta (IL-1β), interleukin-6 (IL-6), and tumor necrosis factor-alpha (TNF-α), are predominantly produced and released from mononuclear cells and macrophages [15]. The three cytokines mentioned above were most frequently studied in relation to the immune-inflammatory response [13]. In this context, numerous publications have reported that CBD was capable of modulating the secretion and function of cytokines from immune-inflammatory cells [12,13,17]. A recent review article by Martini et al. demonstrated that several in vitro and in vivo studies of CBD’s effects on immunomodulation resulted in decreased levels of IL-1β, IL-6, and TNF-α [18]. Furthermore, cannabinoids have demonstrated anti-inflammatory effects on LPS-stimulated human macrophages by reducing the levels of various cytokines [16]. Evidence suggests that epileptic seizures are associated with neuro-inflammatory responses and elevated levels of cytokines, particularly IL-1β, IL-6, and TNF-α [19,20]. The utilization of anti-IL-1, anti-IL-6, and anti-TNF in seizure management has been documented [21,22]. The literature on the impact of anti-seizure medications (ASMs) on cytokine levels is sparse. Nonetheless, some pertinent findings can be examined. Research suggests that gabapentin may modulate TNF-α, IL-1, and IL-6 levels [23], while zonisamide potentially influences TNF-α [24]. Additionally, topiramate has been associated with alterations in IL-6 and IL-1 concentrations [25]. Conversely, phenobarbital, potassium bromide, and levetiracetam showed no evidence or neutral effects on specific cytokines [26]. It is important to note that the immunomodulatory effects of ASMs frequently produce inconsistent and conflicting outcomes, which can be attributed to various confounding factors. As previously noted, CBD possesses anti-inflammatory properties, which may potentially alleviate inflammation in epileptogenesis that could contribute to seizures.

Notwithstanding the aforementioned data, there remains a paucity of knowledge regarding the toxicity and anti-inflammatory effects of CBD on immune cells in companion animals. Consequently, the present study aims to evaluate the cytotoxicity and efficacy of CBD in modulating pro-inflammatory cytokine production, specifically IL-1β, IL-6, and TNF-α, in LPS-stimulated PBMCs in dogs. Furthermore, to investigate the potential clinical implications of CBD on neuroinflammatory responses, an independent study examining CBD co-administered with other anti-seizure medications (ASMs) in epileptic dogs was included.

## 2. Materials and Methods

### 2.1. Ethics

The study was approved by the Committee for the Approval of Animal Care and Use for Scientific Research of the Faculty of Veterinary Medicine, Kasetsart University, Bangkok, Thailand (approval number ACKU 65-VET-062). All experimental procedures and consent forms were conducted in accordance with the institutional fundamental requirements.

### 2.2. Study Design and Procedures

The present investigation consisted of two separate studies. The initial investigation aimed to elucidate the effects of CBD on cytotoxicity and cytokine production in canine PBMCs stimulated with lipopolysaccharide (LPS). The subsequent investigation, conducted independently, explored the potential clinical ramifications of CBD administration on cytokine production by PBMCs isolated from epileptic dogs undergoing concurrent treatment with various ASMs over a 30-day period.

#### 2.2.1. Animal

The first study incorporated six German shepherd dogs, all in good health, with an average age of 3.5 years (ranging from 2 to 6 years) and a mean weight of 26 kg (ranging between 23 and 32 kg). These dogs were sourced from a private farm and individually housed. A minimum 30-day acclimatization period was implemented before blood sampling, during which no medications were administered. At the time of blood collection, all subjects displayed hematological parameters within normal range and exhibited no apparent clinical anomalies.

The second study, constrained by the scarcity of refractory epileptic canines, voluntary participation of owners, and Thai regulations on cannabis usage, was structured as a one-month quasi-experimental single-arm pre/post-treatment analysis. The study involved five dogs diagnosed with refractory epilepsy. To be included, the dogs had to meet certain requirements, including a consistent three-month history of visits to the neurology department prior to enrollment. Additionally, the dogs must have previously undergone magnetic resonance imaging (MRI) and cerebral spinal fluid (CSF) analysis to rule out infectious or structural abnormalities. Furthermore, all participating dogs needed to have experienced a minimum of two seizures per month over a period of at least 12 weeks and be undergoing ongoing treatment with phenobarbital and gabapentin. These subjects were also receiving a minimum of two additional ASMs, such as potassium bromide, levetiracetam, zonisamide, topiramate, and imepitoin. Notably, no steroids or NSAIDs were administered to any of the participants. Following the procurement of signed consent forms from owners, baseline blood samples (PBMCs) were obtained on day 1, prior to CBD treatment initiation. A cautious dosing strategy referred to as a “start-low, go-slow” approach was introduced to the owners. This method emphasizes starting with a low dose and gradually increasing the dosage over time based on the dogs’ tolerability and clinical response. The CBD regimen commenced at 0.5 mg/kg for all included dogs, with individualized dose escalations up to a maximum of 2 mg/kg. On day 30, the dogs underwent re-examination, including routine assessments and post-treatment blood collection.

#### 2.2.2. Canine Blood Sampling and PBMCs Isolation

Whole blood (1–2 mL) was obtained from the donor dogs and collected via the cephalic vein into an anticoagulant tube containing ethylenediaminetetraacetic acid (EDTA). For PBMC isolation, the blood samples were layered on 2 mL of Lymphocyte Separation Medium (LSM) 1.077 g/mL (Corning, NY, USA) and centrifuged at 2000 rpm for 30 min to obtain enriched PBMCs. Following centrifugation, PBMCs were harvested from the interface and washed twice with RPMI-1640 medium (Corning, VA, USA) by centrifugation at 3500 rpm for 10 min. Subsequently, cell count and viability were assessed utilizing the trypan blue exclusion dye assay. Cells exhibiting viability greater than 80% were immediately utilized in subsequent experiments.

#### 2.2.3. Cell Culture

PBMCs were adjusted to 1 × 10^6^ cells/mL and cultured in RPMI-1640 medium containing 2 mM L-glutamine (Corning, VA, USA), 10% heat-inactivated fetal bovine serum (Gibco, Paisley, UK), penicillin (100 U/mL), and streptomycin (100 µg/mL) (Gibco, NY, USA) in a humidified atmosphere of 5% CO_2_ at 37 °C.

#### 2.2.4. Cell Viability of CBD on PBMCs

PBMCs (1 × 10^6^ cells/mL) were seeded into a 96-well plate and pre-incubated for 1–2 h in a humidified atmosphere with 5% CO_2_ at 37 °C. Subsequently, both cell types were treated with various concentrations of CBD in 0.5% ethanol (2, 4, 6, 8, 10, 12, 14, 16, 18, and 20 µg/mL). The cells were then incubated for 24 h for PBMCs. Following treatment, cell viability was assessed using the CCK-8 colorimetric assay. In brief, 10 µL of CCK-8 solution (Dojindo, Kumamoto, Japan) was added to each well containing both cell types, and the plates were incubated for 2–4 h. The absorbance of each well was subsequently measured at 450 nm using a microplate reader (EnSight^®^ Multimode plate reader, PerkinElmer, Waltham, MA, USA). Cell viability was determined by calculating the percentage of absorbance relative to the control group (non-treated cells).

#### 2.2.5. Cell Viability of CBD on LPS-Stimulated PBMCs

PBMCs (1 × 10^6^ cells/mL) were seeded into a 96-well plate and pre-incubated for 1–2 h in a humidified atmosphere with 5% CO_2_ at 37 °C. Subsequently, the cells were treated with various concentrations of CBD (2, 4, 6, 8, 10, 12, 14, 16, 18, and 20 µg/mL) and DEX (Dexamethasone) as a positive drug control (5 µg/mL) (Lodexa, Bangkok, Thailand) in the absence or presence of LPS (1 µg/mL) (Sigma-Aldrich, St. Louis, MO, USA) and then incubated for 24 h. After the treatment, a CCK-8 colorimetric assay was performed.

#### 2.2.6. Determination of IL-1β, IL-6 and TNF-α Release

PBMCs (1 × 10^6^ cells/mL) were seeded into a 96-well plate and pre-incubated for 1–2 h in a humidified atmosphere with 5% CO_2_ at 37 °C. Subsequently, the cells were treated with concentrations of CBD (5, 7.5, 10, 15, and 30 µg/mL) and DEX (5 µg/mL) in the presence of LPS (1 µg/mL) and then incubated for 24 h. Following incubation, the supernatants of each well were collected by centrifugation at 3500 rpm for 10 min and stored at −80 °C until analysis. The levels of pro-inflammatory cytokine release were quantified using commercial IL-1β, IL-6, and TNF-α ELISA kits (Abcam, Waltham, MA, USA). For blood samples from epileptic dogs, PBMCs were isolated within 12 h of sample receipt from the unit. Subsequently, cell viability assays and ELISA tests were conducted following the protocol.

### 2.3. Statistical Analysis

The data obtained from the research are presented as mean ± standard deviation (S.D.), with each experiment replicated at least twice to ensure reliability. Statistical analysis of the data was conducted to compare differences between groups using the Kruskal-Wallis test followed by Dunn’s multiple comparison test to determine statistical significance. A significance level of 95% confidence was established, with *p* < 0.05 considered statistically significant. The statistical analysis of cytokine levels in the second study was performed using the Wilcoxon test at a 95% confidence level (*p* < 0.05). All analyses were performed using GraphPad Prism version 9 (GraphPad Software, LLC, Boston, MA, USA).

## 3. Results

### 3.1. Isolation and Optimization of PBMC Culture Conditions from Canine Blood Samples

The cell viability at 0 h was 98.68 ± 0.26%, and after 24 h, the viability decreased to 97.37 ± 0.20%. This reduction in viability over 24 h was statistically significant (*p* < 0.01) but remained within an acceptable range (≥80%) according to ISO 10993-5:2009 [27].

**Figure 1:**
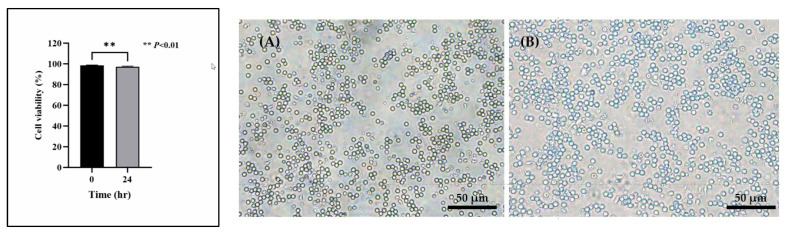
The viability of PBMCs isolated from canine blood samples was assessed at 0 h (**A**) and 24 h (**B**) utilizing the Trypan Blue Exclusion Assay (*n* = 6). The results are presented as mean ± S.D. A statistically significant difference in viability was observed between the 0 and 24 h time points, with a confidence level of 99% (*p* < 0.01).

As illustrated in Figure 1, after 24 h, the morphology of the PBMCs exhibited minor alterations; however, the majority of the cells maintained a spherical shape, with no significant indications of clustering or aggregation when compared to the 0-h time point. Consequently, the culture conditions and time period of 0 to 24 h were deemed suitable for PBMC studies, ensuring that the cell quality did not adversely affect subsequent experimental procedures.

### 3.2. LPS-Stimulated PBMCs Cytotoxicity

**Figure 2:**
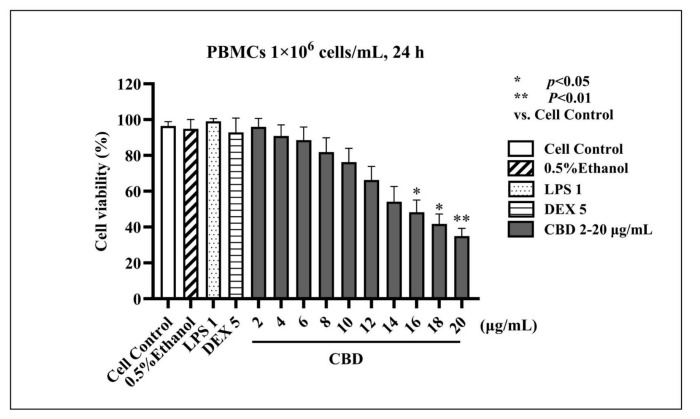
The viability assessment results of PBMCs (×10^6^ cells/mL) from canines when evaluated with CBD concentrations ranging from 2–20 µg/mL, 0.5% ethanol, 1 µg/mL LPS, and 5 µg/mL DEX for 24 h (mean ± S.D., *n* = 6). Ethanol at concentrations of 0.5% exhibited no cytotoxicity in cell proliferation in vitro. The distinct asterisks above each dilution indicate the statistical significance of that dilution (*p* < 0.01), while identical asterisks above dilutions denote no statistical significance.

**Figure 3:**
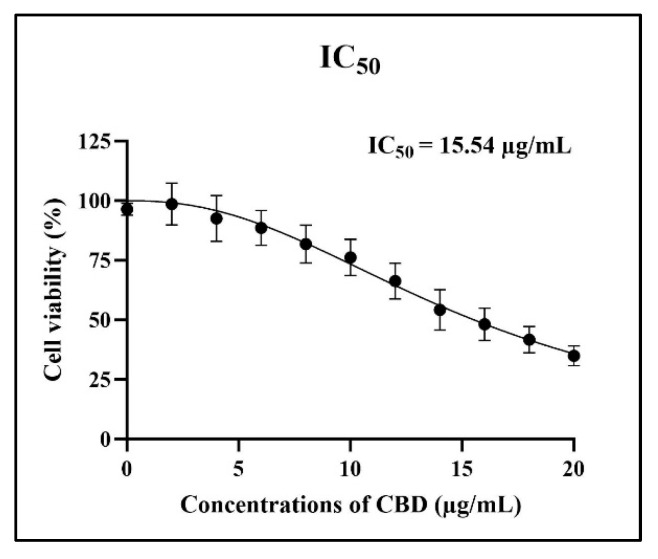
Percentage of PBMCs viability assay. Cell viability of PBMCs from canines when exposed to cannabidiol (CBD) concentrations ranging from 2–20 µg/mL for 24 h (mean ± S.D.).

PBMCs isolated from canine blood samples were subjected to CBD treatment at concentrations ranging from 2–20 µg/mL for 24 h, and cell viability was assessed using the CCK-8 colorimetric assay. The results indicated that CBD concentrations between 2–14 µg/mL exhibited no significant cytotoxicity to PBMCs when compared to the control group (Figure 2), with viability rates exceeding 80%. The dose-response curve for CBD-mediated inhibition of cell activity at 50% (half maximal inhibitory concentration; IC_50_) was calculated to be 15.54 µg/mL, as illustrated in Figure 3.

### 3.3. Pro-Inflammatory Cytokines in LPS-Stimulated PBMCs Affected by CBD

To investigate the anti-inflammatory properties of CBD, this study examined the effects of CBD on three major pro-inflammatory cytokines, IL-1β, IL-6, and TNF-α, from LPS-stimulated PBMCs in dogs. After assessing the viability of LPS-stimulated PBMCs with CBD at concentrations of 5, 7.5, 10, 15, and 30 µg/mL and DEX at concentrations of 5 µg/mL, it was determined that CBD at concentrations of 5–10 µg/mL and DEX at concentrations of 5 µg/mL did not induce cell toxicity.

**Figure 4:**
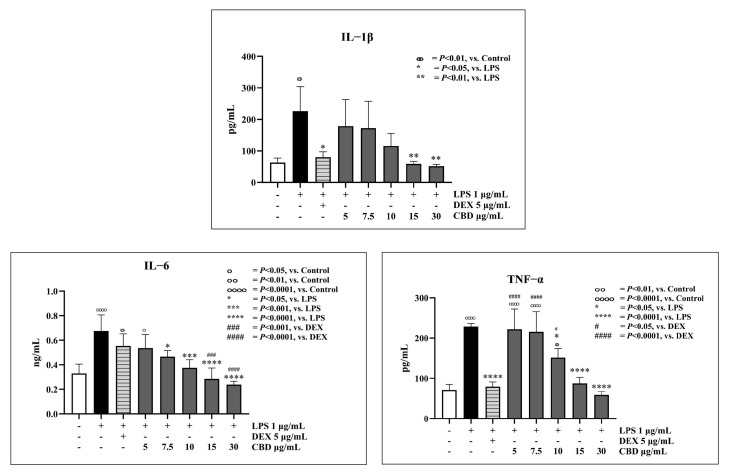
Pro-inflammatory cytokine secretion (IL-1β, IL-6, and TNF-α) from LPS-stimulated canine PBMCs co-cultured with CBD at concentrations of 1, 7.5, 15, and 30 µg/mL and DEX (dexamethasone) at a concentration of 5 µg/mL (mean ± S.D.). Different symbols above each bar indicate statistically significant differences between groups (*p* < 0.01), while identical symbols denote no significant difference.

The IL-1β from canine LPS-stimulated PBMCs co-cultured with CBD at a concentration range of 5–10 µg/mL exhibited a statistically insignificant decrease in secretion when compared to the control. Upon comparison of the ability to reduce TNF-α secretion between LPS-stimulated PBMCs and those co-cultured with CBD at a concentration of 5–7.5 µg/mL, no statistically significant difference was observed. CBD at a concentration started from 7.5 µg/mL demonstrated a statistically significant reduction in the secretion of IL-6. The effect of CBD was most pronounced on IL-6 activity. As depicted in Figure 4, this finding elucidated the immunomodulatory activity of CBD, which appears to be in a dose-dependent manner by reducing the production of pro-inflammatory cytokines induced by LPS stimulation in canine PBMCs.

### 3.4. Pro-Inflammatory Cytokines Modulation in Refractory Epileptic Dogs Co-Treated with CBD

Despite the number of epileptic dogs that presented at the animal hospital, only five animals suffering from refractory epilepsy met the inclusion criteria. However, in the cases of IL-1β and IL-6, only four dogs were included in the investigation due to insufficient PBMC samples. At day 0, all included dogs exhibited normal ranges in hematology and blood chemistry tests, with the exception of elevated ALK.

As a result, the mean IL-1β secretion levels from PBMCs on day 0 and day 30 were 119.6 and 77.41 pg/mL, respectively. It is noteworthy that one dog subject exhibited markedly elevated IL-1β levels on the initial day, while two of the four dogs demonstrated comparable IL-1β levels between the two examination days. IL-6 levels in four dogs were elevated on day 0 compared to day 30, with mean values of 0.725 ng/mL and 0.525 ng/mL, respectively. No statistically significant difference was observed between the time points for either IL-1β and IL-6.

**Figure 5:**
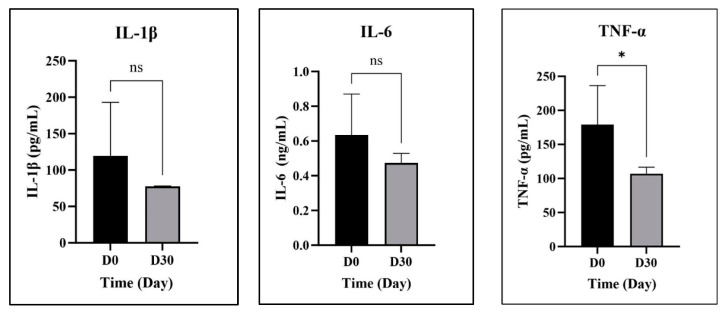
Pro-inflammatory cytokine secretion (IL-1β, IL-6, and TNF-α) from PBMCs of canine refractory epilepsy subjects co-treated with CBD for 30 days (mean ± S.D.). Asterisk denotes statistical significance (*p* < 0.05), and “ns” indicates non-significant results.

Regarding TNF-α, elevated levels on day 0 compared to day 30 were observed in all five participating dogs. Following co-treatment with CBD, a significantly lower level of TNF-α was observed at day 30 (106.99 pg/mL) compared to day 0 (179.05 pg/mL). Figure 5 depicts the production of pro-inflammatory cytokines (IL-1β, IL-6, and TNF-α) by PBMCs obtained from dogs diagnosed with refractory epilepsy, following a 30-day co-administration of CBD.

## 4. Discussion

### 4.1. Cytotoxic Effect of CBD on LPS-Stimulated PBMCs

Cannabinoids are chemical compounds, primarily produced by the *Cannabis sativa* plant, that reportedly interact with the endocannabinoid system and exert various biological effects. Among these, CBD is of particular interest and is being investigated for its potential use in treating various conditions, such as pain, inflammation, and neurological disorders in animals [7,8,11].

The pro-inflammatory cytokines, including IL-1β, IL-6, and TNF-α, most commonly serve as markers to study immune-inflammatory responses [13]. Consequently, this current study established a model for evaluating the cytotoxicity and efficacy of CBD in modulating pro-inflammatory cytokine production after the co-culture of isolated PBMCs from healthy dogs with LPS.

Studies have been conducted utilizing LPS-stimulated PBMCs to evaluate cytokine secretion, as measured by ELISA [5,28,29]. The kinetics of pro-inflammatory cytokine production in LPS-stimulated human PBMCs have been reported to indicate the time-course effect of peak cytokine secretion: IL-1β and TNF-α at 12 h, with IL-6 at 20 h [30]. Consequently, in this study, the decision was made to culture PBMCs from LPS-stimulated blood dog samples for 24 h, as cell viability remained unaltered and this duration was sufficient for LPS-stimulated PBMCs to secrete pro-inflammatory cytokines.

Our results confirmed that LPS at 1 µg/mL acts as an inflammatory stimulus to induce IL-1β, IL-6, and TNF-α production in canine PBMCs [31]. Similarly, PBMCs were found to be more sensitive than whole blood when stimulated with LPS [32]. Therefore, these data indicate an increase in pro-inflammatory cytokine production after LPS stimulation. Certain evidence suggests that LPS could modulate cannabinoid receptor expression in an inconsistent manner, both up-regulating and down-regulating receptor expression [33]. However, several factors with complex mechanisms are involved in cannabinoid receptor expression, which are not yet clearly elucidated.

To the best of the authors’ knowledge, this is the first study investigating the possible immunomodulatory response in terms of cytokine patterns, toxicity, and effects of CBD on LPS-stimulated canine PBMCs. The evaluation of CBD’s effect on cell viability in this investigation revealed that CBD concentrations ranging from 2–14 µg/mL showed no effect on the viability of PBMCs when using a cut point of living cells greater than 80%. Indeed, the current results provided a calculated IC_50_ of 15.54 µg/mL.

Although limited data exist on CBD-affected cell viability in target animals other than laboratory rodents, the current results align with previous studies in target animals. For instance, CBD at concentrations ranging from 2.5 to 10 μg/mL was found to markedly inhibit the proliferation of canine cancer cells [34]. Another study on the toxicity of CBD on PBMC cells in horses demonstrated that CBD affected cell viability at a concentration range of 6–10 μg/mL [35]. However, these concentrations, including those in the present study, are often considered to exceed normal physiological ranges. Consistent findings from canine studies have demonstrated that plasma CBD concentrations typically range from approximately 0.05–0.5 μg/mL for doses between 1–10 mg/kg [36,37,38,39]. Additionally, when hemp extract rich in CBD is administered, the primary compound detected is CBDA, not CBD, with both substances generally found in comparable concentrations [38,40].

Recently, an experiment on CBD toxicity in human PBMC cells demonstrated that CBD at concentrations of 40 and 80 μM exhibited toxicity effects, while 20 μM CBD displayed variable toxicity effects on PBMCs, depending on the individual [41]. Similarly, regarding individual responses, Brown and colleagues evaluated the effect of CBD and THC on cytokine protein expression in canine PBMCs and suggested that cannabinoid effects may be exhibited in a dog-dependent manner [12]. This indicated that the role of cannabinoids on immunomodulatory properties was substantially complex, with multiple influencing factors, such as animal species, dosing, cell type, health status, and the timeframe of action [7,12,42].

### 4.2. Effect of CBD on Pro-Inflammatory Cytokines in LPS-Stimulated PBMCs

Clinically, several reports have demonstrated that CBD alleviated the symptoms of canines suffering from inflammation, suggesting CBD was associated with cannabinoid-mediated pro- and anti-inflammatory cytokine production [7,10].

A previous systematic review of in vivo studies on the effects of cannabinoids on pro- and anti-inflammatory cytokines indicated that the three most commonly studied cytokines were IL-1β, IL-6, and TNF-α [13] In summary of the literature, the majority of investigations found that CBD reduced IL-1β, IL-6, and TNF-α levels, with one exception reporting an increase in IL-6 levels [13].

Our findings clearly demonstrated that CBD has been predominantly implicated in reducing IL-6 secretion, beginning at the initial dose (7.5 μg/mL). The results from this study correlated with a previous report that confirmed that IL-6 was considered a sensitive marker cytokine in response to CBD [16]. This aligns with a recent study suggesting that IL-6, unlike IL-1β and TNF-α, could be a useful marker of peripheral inflammation in elderly humans [43]. Additionally, all tested cytokines in our study exhibited a tendency to decrease in a dose-dependent manner. It is noteworthy that the significant reduction of cytokines at higher doses of CBD might be attributed to a reduction in cell viability. CBD-exhibited biological effects and CBD-induced cytotoxicity increasing in a dose- and time-dependent manner have been previously described [14,44]. Moreover, at higher concentrations, CBD may overwhelm the body’s cellular defense mechanisms, triggering oxidative stress, apoptosis, mitochondrial dysfunction, and other forms of cellular toxicity, ultimately leading to reduced cell viability in PBMCs [14,45].

Numerous studies have demonstrated the potential of CBD to alter inflammatory cytokines; however, most of these investigations were conducted in laboratory animals or human-derived cells [16,18]. To date, research on both in vivo and ex vivo studies of CBD modulating inflammatory cells in companion animals is limited. A small number of studies have reported the effect of CBD on inflammatory cytokine responses in target animals, such as dogs. Among these, Gugliandolo et al. demonstrated an ex vivo model of LPS-stimulated whole blood from dogs using CBD at 50 and 100 μg/mL, which decreased IL-6 and TNF-α levels and reduced Nf-kB and COX-2 expression. However, the results showed no effect of CBD on IL-10 levels. This publication provided the first description of CBD’s effects on cytokine production directly in dogs’ immune cells from whole blood, without data related to cytotoxicity [46]. Nevertheless, due to differences in study design and models, it is inappropriate to directly compare the dose and effectiveness of CBD among these reports. It is worth noting that although all participating dogs in this project were healthy with normal hematological profiles, other undetected internal pathological conditions might have existed. In this regard, we concur with the explanation provided by Brown et al., suggesting that studies using a single breed of dog and health status with the presence of various disease states might influence the immune response to CBD [12].

In addition, it has been reported that CBD selectively suppressed the secretion of IL-1β and IL-6, while TNF-α showed no effect or increased secretion by human monocytes activated through most toll-like receptors (TLRs). However, it is suggested that an alteration in pro-inflammatory cytokines, such as IL-1β and IL-6, by CBD may depend on health status and pathological conditions [47]. In this context, it is important to note that CBD demonstrates anti-inflammatory properties through multiple mechanisms, one of which involves the activation of peroxisome proliferator-activated receptors (PPARs), particularly PPARγ. PPARs influence immune regulation and cytokine expression through complex mechanisms, including alterations in gene transcription and translation [48]. These effects often require extended periods to manifest fully. Consequently, acute studies (e.g., 24-h in vitro assays) may not capture the full extent of PPAR effects. However, our results provide evidence that corroborates previous findings regarding the cytokines affected by CBD.

There are shortcomings in this study that should be noted. First, there was a limited sample size in terms of the breed and number of dogs used. Additionally, there was a narrow range of CBD doses used in the experiment with LPS-stimulated PBMCs to study the secretion of pro-inflammatory cytokines, which may have resulted in insufficient data points to elucidate the response of immunomodulation. This ex vivo experimental design may not directly reflect how CBD affects immune response in vivo or under multi-factor environments. Collectively, these limitations warrant further in-depth and broad mechanistic studies conducted under different physio-pathological conditions, using various animal models and CBD dosages.

### 4.3. Cytokines Alteration in PBMCs of Refractory Epileptic Dogs Co-Treated with CBD

The approval of Epidiolex by the FDA in 2018 for pediatric seizure disorders has led to increased interest in the application of CBD as a therapeutic approach for epileptic conditions [49]. Although the mechanism and efficacy of CBD on anti-seizure activity remain unclear, its anti-inflammatory effects may play a crucial role in neuroprotective activity [50]. Despite limited information regarding the use of CBD in epileptic dogs, anecdotal evidence of this application has been observed. Specifically, there are few studies on the application of CBD with and without other phytocannabinoids to control seizures in epileptic dogs [51,52,53]. Recently, Potschka and colleagues reviewed the potential use of CBD in the treatment of canine epilepsy [11].

There has been evidence that the alteration of the proinflammatory response may affect epileptogenesis [20,54,55]. Notably, prior studies have demonstrated that pro-inflammatory cytokines, such as IL-1β, IL-6, and TNF-α, are markedly increased in patients with refractory repetitive acute seizures and are associated with seizure activity [19,20]. All three main cytokines have reportedly been involved in CNS inflammatory response and epileptogenesis [22]. Although epileptogenesis can enhance blood-brain barrier (BBB) permeability, the interplay between epileptogenesis, BBB permeability, and cytokines is complex and multifaceted [56]. Moreover, limited information on neuroinflammation in canine epileptogenesis is available [20]. Among neuro-inflammatory cytokines, evidence of increased IL-1β levels in serum [57] and IL-6 and TNF-α levels in CSF [58] of epileptic dogs has been reported.

In the current study, five refractory epilepsy dogs were included. IL-1β in the serum of epileptic dogs in our current results was found at 119.61 pg/mL, which was comparable to the previous report by Kostic (118.6 ± 81.4 pg/mL) [57]. The results indicated that average levels of IL-1β and IL-6 tended to decrease over time following CBD ingestion. However, there was no statistically significant difference in both cytokines between pre- and post-investigation. Regarding anti-cytokine agents for the treatment of epilepsy, the efficacy of using anti-IL-1 and anti-IL-6 to control seizures in drug-resistant epilepsy and refractory status epilepticus has been reviewed [22].

Previous reports have indicated that TNF-α and IL-1β play important roles in neuromodulation, which may affect seizure susceptibility in animal models [59]. Notably, our findings demonstrated that TNF-α was significantly reduced after dogs received CBD. This aligns with earlier reports suggesting that the use of anti-TNF-α agents may be beneficial in resisting seizures in certain epileptic disorders [21].

In accordance with Arulsamy and Shaikh (2020), consistent improvement in seizure activity was achieved by downregulating TNF-α expression compared with other pro-inflammatory cytokines. This may be attributed to TNF-α reducing the expression of surface gamma-aminobutyric acid A receptors (GABAA), leading to a decrease in inhibitory synapse activity [60]. However, there is a paucity of literature reporting the use of anti-TNF agents in epilepsy. Consequently, the role and mechanism of TNF-α in epileptogenesis have not been fully elucidated [22,55]. As mentioned earlier, the administration of ASMs in epilepsy management has been observed to influence cytokine levels, albeit with considerable variability and inconsistency [23,24,25,26]. This diversity in results can be attributed to a complex interplay of factors influencing both drug efficacy and research methodologies.

Taken together, in cases of epileptic dogs, CBD may have the potential to counteract pro-inflammatory cytokines, particularly TNF-α. This finding may be valuable in exploring specific targeting of inflammatory mediator pharmaceuticals to treat canine epilepsy, and the current findings may augment its potential as an adjunctive therapy to conventional ASMs. However, the extent of cytokine modulation may depend on the type of epilepsy, severity, state of disease, timeframe of inflammation (acute-chronic), and pharmacokinetics and pharmacodynamics of cannabinoids. As previously discussed regarding cytokines regulated by PPARs, it is important to emphasize the significance of chronic dosing regimens, as demonstrated in the epileptic dogs, which could potentially modulate the immune response.

A significant limitation of this independent study was the low statistical power due to the small sample size of volunteer participants. An additional limitation arose from the individualized CBD administration following the “start-low go-slow” principle (range of 0.5–2 mg/kg) for each dog, resulting in varied CBD dosages during treatment. Furthermore, the diversity in the state (disease progression) and severity of the disease in each dog necessitated different ASMs, which may have influenced the immunomodulatory and clinical outcomes. Nevertheless, we posit that this quasi-experimental study provides the first evidence indicating that TNF-α was the predominant cytokine affected by CBD, which may serve as an important target in the treatment of refractory canine epilepsy. Given the current context, additional investigation is necessary to examine cytokine levels in future studies, aiming to elucidate whether the effects are localized to the brain, systemic, or encompass both domains. Considering the limitations of this study, further research is warranted to corroborate these preliminary results and their potential therapeutic applications.

## 5. Conclusions

Conclusively, the key finding of the current study demonstrates that cannabidiol (CBD), a major non-psychoactive phytocannabinoid constituent from hemp, attenuates the production and release of selected pro-inflammatory cytokines from lipopolysaccharide (LPS)-activated peripheral blood mononuclear cells (PBMCs) in dogs and exhibits a tendency to down-regulate the immunomodulation towards a specific inflammatory response in epileptic dogs.

## Data Availability

Data are contained within the article.

## References

[1] Martinez Naya N., Kelly J., Corna G., Golino M., Abbate A., Toldo S. (2023). Molecular and Cellular Mechanisms of Action of Cannabidiol. Molecules.

[2] Silver R.J. (2019). The endocannabinoid system of animals. Animals.

[3] Donvito G., Nass S.R., Wilkerson J.L., Curry Z.A., Schurman L.D., Kinsey S.G., Lichtman A.H. (2018). The Endogenous Cannabinoid System: A Budding Source of Targets for Treating Inflammatory and Neuropathic Pain. Neuropsychopharmacology.

[4] Bilbao A., Spanagel R. (2022). Medical cannabinoids: A pharmacology-based systematic review and meta-analysis for all relevant medical indications. BMC Med..

[5] Furgiuele A., Marino F., Rasini E., Legnaro M., Luini A., Albizzati M.G., di Flora A., Pacchetti B., Cosentino M. (2023). Effect of Cannabidiol on Human Peripheral Blood Mononuclear Cells and CD4 + T Cells. Int. J. Mol. Sci..

[6] Andre C.M., Hausman J.-F., Guerriero G. (2016). Cannabis sativa: The plant of the thousand and one molecules. Front. Plant Sci..

[7] Corsato Alvarenga I., Panickar K.S., Hess H., McGrath S. (2023). Scientific validation of Cannabidiol for management of dog and cat diseases. Annu. Rev. Anim. Biosci..

[8] R C Coelho M.P., de O P Leme F., A Moreira F., E M T Branco S., M Melo M., G de Melo E. (2021). Current review of hemp-based medicines in dogs. J. Vet. Pharmacol. Ther..

[9] Williams N.N.B., Ewell T.R., Abbotts K.S.S., Harms K.J., Woelfel K.A., Dooley G.P., Weir T.L., Bell C. (2021). Comparison of five Oral Cannabidiol preparations in adult humans: Pharmacokinetics, body composition, and heart rate variability. Pharmaceuticals.

[10] Gamble L.J., Boesch J.M., Frye C.W., Schwark W.S., Mann S., Wolfe L., Brown H., Berthelsen E.S., Wakshlag J.J. (2018). Pharmacokinetics, Safety, and Clinical Efficacy of Cannabidiol Treatment in Osteoarthritic Dogs. Front. Vet. Sci..

[11] Potschka H., Bhatti S.F.M., Tipold A., McGrath S. (2022). Cannabidiol in canine epilepsy. Vet. J..

[12] Brown C., Mitsch M., Blankenship K., Campbell C., Pelanne M., Sears J., Bell A., Olivier A.K., Ross M.K., Archer T. (2023). Canine immune cells express high levels of CB1 and CB2 cannabinoid receptors and cannabinoid-mediated alteration of canine cytokine production is vehicle-dependent. Vet. Immunol. Immunopathol..

[13] Henshaw F.R., Dewsbury L.S., Lim C.K., Steiner G.Z. (2021). The effects of cannabinoids on pro- and anti-inflammatory cytokines: A systematic review of in vivo studies. Cannabis Cannabinoid Res..

[14] Pagano S., Coniglio M., Valenti C., Federici M.I., Lombardo G., Cianetti S., Marinucci L. (2020). Biological effects of Cannabidiol on normal human healthy cell populations: Systematic review of the literature. Biomed. Pharmacother..

[15] Elgellaie A., Thomas S.J., Kaelle J., Bartschi J., Larkin T. (2023). Pro-inflammatory cytokines IL-1α, IL-6 and TNF-α in major depressive disorder: Sex-specific associations with psychological symptoms. Eur. J. Neurosci..

[16] Zaiachuk M., Suryavanshi S.V., Pryimak N., Kovalchuk I., Kovalchuk O. (2023). The Anti-Inflammatory Effects of Cannabis sativa Extracts on LPS-Induced Cytokines Release in Human Macrophages. Molecules.

[17] Kent-Dennis C., Klotz J.L. (2023). Immunomodulation by cannabidiol in bovine primary ruminal epithelial cells. BMC Vet. Res..

[18] Martini S., Gemma A., Ferrari M., Cosentino M., Marino F. (2023). Effects of Cannabidiol on Innate Immunity: Experimental Evidence and Clinical Relevance. Int. J. Mol. Sci..

[19] Arulsamy A., Shaikh M.F. (2020). Tumor Necrosis Factor-α, the Pathological Key to Post-Traumatic Epilepsy: A Comprehensive Systematic Review. ACS Chem. Neurosci..

[20] von Rüden E.L., Potschka H., Tipold A., Stein V.M. (2023). The role of neuroinflammation in canine epilepsy. Vet. J..

[21] Michev A., Orsini A., Santi V., Bassanese F., Veraldi D., Brambilla I., Marseglia G.L., Savasta S., Foiadelli T. (2022). An Overview of The Role of Tumor Necrosis Factor-Alpha in Epileptogenesis and Its Terapeutic Implications. Acta Biomed. Atenei Parm..

[22] Costagliola G., Depietri G., Michev A., Riva A., Foiadelli T., Savasta S., Bonuccelli A., Peroni D., Consolini R., Marseglia G.L. (2022). Targeting Inflammatory Mediators in Epilepsy: A Systematic Review of Its Molecular Basis and Clinical Applications. Front. Neurol..

[23] Lee B.S., Jun I.G., Kim S.H., Park J.Y. (2013). Intrathecal gabapentin increases interleukin-10 expression and inhibits pro-inflammatory cytokine in a rat model of neuropathic pain. J. Korean Med. Sci..

[24] Hossain M.M., Weig B., Reuhl K., Gearing M., Wu L.J., Richardson J.R. (2018). The anti-parkinsonian drug zonisamide reduces neuroinflammation: Role of microglial Na_(v)_ 1.6. Exp. Neurol..

[25] Andrzejczak D., Woldan-Tambor A., Bednarska K., Zawilska J.B. (2016). The effects of topiramate on lipopolysaccharide (LPS)-induced proinflammatory cytokine release from primary rat microglial cell cultures. Epilepsy Res..

[26] Guenther S., Bauer S., Hagge M., Knake S., Olmes D.G., Tackenberg B., Rosenow F., Hamer H.M. (2014). Chronic valproate or levetiracetam treatment does not influence cytokine levels in humans. Seizure.

[27] (2009). Biological Evaluation of Medical Devices—Part 5: Tests for In Vitro Cytotoxicity.

[28] Wu C.-Y., Fan W.-L., Chiu Y.-M., Yang H.-Y., Lee W.-I., Huang J.-L. (2021). Lipopolysaccharide stimulation test on cultured PBMCs assists the discrimination of cryopyrin-associated periodic syndrome from systemic juvenile idiopathic arthritis. Sci. Rep..

[29] Fitzpatrick J.-M., Hackett B., Costelloe L., Hind W., Downer E.J. (2022). Botanically-Derived D9-Tetrahydrocannabinol and Cannabidiol, and Their 1:1 Combination, Modulate Toll-like Receptor 3 and 4 Signalling in Immune Cells from People with Multiple Sclerosis. Molecules.

[30] Kumolosasi E., Salim E., Jantan I., Ahmad W. (2014). Kinetics of Intracellular, Extracellular and Production of Pro-Inflammatory Cytokines in Lipopolysaccharide- Stimulated Human Peripheral Blood Mononuclear Cells. Trop. J. Pharm. Res..

[31] Silva S., Ganguly K., Fresquez T.M., Gupta G., McCleskey T.M., Chaudhary A. (2009). Beryllium alters lipopolysaccharide-mediated intracellular phosphorylation and cytokine release in human peripheral blood mononuclear cells. J. Occup. Environ. Hyg..

[32] Schildberger A., Rossmanith E., Eichhorn T., Strassl K., Weber V. (2013). Monocytes, peripheral blood mononuclear cells, and THP-1 cells exhibit different cytokine expression patterns following stimulation with lipopolysaccharide. Mediat. Inflamm..

[33] Klein T.W. (2005). Cannabinoid-based drugs as anti-inflammatory therapeutics. Nat. Rev. Immunol..

[34] Henry J.G., Shoemaker G., Prieto J.M., Hannon M.B., Wakshlag J.J. (2021). The effect of cannabidiol on canine neoplastic cell proliferation and mitogen-activated protein kinase activation during autophagy and apoptosis. Vet. Comp. Oncol..

[35] Turner S., Barker V.D., Adams A.A. (2021). Effects of Cannabidiol on the In Vitro Lymphocyte Pro-Inflammatory Cytokine Production of Senior Horses. J. Equine Vet. Sci..

[36] Hartsel J.A., Boyar K., Pham A., Silver R.J., Makriyannis A., Gupta R.C., Srivastava A., Lall R. (2019). Cannabis in Veterinary Medicine: Cannabinoid Therapies for Animals. Nutraceuticals in Veterinary Medicine.

[37] Tittle D., Wakshlag J., Schwark W., Lyubimov A., Zakharov A., Gomez B. (2022). Twenty-Four Hour and One-Week Steady State Pharmacokinetics of Cannabinoids in Two Formulations of Cannabidiol and Cannabidiolic Acid Rich Hemp in Dogs. Med. Res. Arch..

[38] Schwark W.S., Wakshlag J.J. (2023). A One Health perspective on comparative cannabidiol and cannabidiolic acid pharmacokinetics and biotransformation in humans and domestic animals. Am. J. Vet. Res..

[39] Limsuwan S., Phonsatta N., Panya A., Asasutjarit R., Tansakul N. (2024). Pharmacokinetics behavior of four cannabidiol preparations following single oral administration in dogs. Front. Vet. Sci..

[40] Wakshlag J.J., Schwark W.S., Deabold K.A., Talsma B.N., Cital S., Lyubimov A., Iqbal A., Zakharov A. (2020). Pharmacokinetics of Cannabidiol, Cannabidiolic Acid, Delta9-Tetrahydrocannabinol, Tetrahydrocannabinolic Acid and Related Metabolites in Canine Serum After Dosing with Three Oral Forms of Hemp Extract. Front. Vet. Sci..

[41] Jindaphun K., Takheaw N., Laopajon W., Pata S., Kasinrerk W. (2023). Toxicity effects of Cannabidiol (CBD) on immune cells. J. Assoc. Med. Sci..

[42] Massi P., Vaccani A., Parolaro D. (2006). Cannabinoids, immune system and cytokine network. Curr. Pharm. Des..

[43] Tylutka A., Walas Ł., Zembron-Lacny A. (2024). Level of IL-6, TNF, and IL-1β and age-related diseases: A systematic review and meta-analysis. Front. Immunol..

[44] Choi Park W.-H.-D., Baek S.-H., Chu J.-P., Kang M.-H., Mi Y.-J. (2008). Cannabidiol Induces Cytotoxicity and Cell Death via Apoptotic Pathway in Cancer Cell Lines. Biomol. Ther..

[45] Jantas D., Leskiewicz M., Regulska M., Procner M., Warszynski P., Lason W. (2024). Protective Effects of Cannabidiol (CBD) against Qxidative Stress, but Not Excitotoxic-Related Neuronal Cell Damage—An In Vitro Study. Biomolecules.

[46] Gugliandolo E., Licata P., Peritore A.F., Siracusa R., D’Amico R., Cordaro M., Fusco R., Impellizzeri D., Di Paola R., Cuzzocrea S. (2021). Effect of Cannabidiol (CBD) on Canine Inflammatory Response: An Ex Vivo Study on LPS Stimulated Whole Blood. Vet. Sci..

[47] Sermet S., Li J., Bach A., Crawford R.B., Kaminski N.E. (2021). Cannabidiol selectively modulates interleukin (IL)-1β and IL-6 production in toll-like receptor activated human peripheral blood monocytes. Toxicology.

[48] O’Sullivan S.E. (2016). An update on PPAR activation by cannabinoids. Br. J. Pharmacol..

[49] Abu-Sawwa R., Scutt B., Park Y. (2020). Emerging Use of Epidiolex (Cannabidiol) in Epilepsy. J. Pediatr. Pharmacol. Ther..

[50] Silvestro S., Schepici G., Bramanti P., Mazzon E. (2020). Molecular Targets of Cannabidiol in Experimental Models of Neurological Disease. Molecules.

[51] McGrath S., Bartner L.R., Rao S., Packer R.A., Gustafson D.L. (2019). Randomized blinded controlled clinical trial to assess the effect of oral cannabidiol administration in addition to conventional antiepileptic treatment on seizure frequency in dogs with intractable idiopathic epilepsy. J. Am. Vet. Med. Assoc..

[52] Garcia G.A., Kube S., Carrera-Justiz S., Tittle D., Wakshlag J.J. (2022). Safety and efficacy of cannabidiol-cannabidiolic acid rich hemp extract in the treatment of refractory epileptic seizures in dogs. Front. Vet. Sci..

[53] Rozental A.J., Weisbeck B.G., Corsato Alvarenga I., Gustafson D.L., Kusick B.R., Rao S., Bartner L.R., McGrath S. (2023). The efficacy and safety of cannabidiol as adjunct treatment for drug-resistant idiopathic epilepsy in 51 dogs: A double-blinded crossover study. J. Vet. Intern. Med..

[54] Balosso S., Maroso M., Sanchez-Alavez M., Ravizza T., Frasca A., Bartfai T., Vezzani A. (2008). A novel non-transcriptional pathway mediates the proconvulsive effects of interleukin-1beta.Brain. A J. Neurol..

[55] Vezzani A., Friedman A., Dingledine R.J. (2013). The role of inflammation in epileptogenesis. Neuropharmacology.

[56] Löscher W., Friedman A. (2020). Structural, Molecular, and Functional Alterations of the Blood-Brain Barrier during Epileptogenesis and Epilepsy: A Cause, Consequence, or Both?. Int. J. Mol. Sci..

[57] Kostic D., Carlson R., Henke D., Rohn K., Tipold A. (2019). Evaluation of IL-1β levels in epilepsy and traumatic brain injury in dogs. BMC Neurosci..

[58] Merbl Y., Sommer A., Chai O., Aroch I., Zimmerman G., Friedman A., Soreq H., Shamir M.H. (2014). Tumor necrosis factor-α and interleukin-6 concentrations in cerebrospinal fluid of dogs after seizures. J. Vet. Intern. Med..

[59] Balosso S., Ravizza T., Aronica E., Vezzani A. (2013). The dual role of TNF-α and its receptors in seizures. Exp. Neurol..

[60] Olmos G., Lladó J. (2014). Tumor necrosis factor alpha: A link between neuroinflammation and excitotoxicity. Mediat. Inflamm..

